# Federal 340B Program Payment Scheme for Drugs Designated As Orphan Products: Congressional Clarification Needed to Close the Government-Industry Revolving Door

**DOI:** 10.1200/JCO.2016.68.2989

**Published:** 2016-10-03

**Authors:** Y. Tony Yang, Brian Chen, Charles L. Bennett

**Affiliations:** Y. Tony Yang, George Mason University, Fairfax, VA; and Brian Chen and Charles L. Bennett, University of South Carolina, Columbia, SC

Drugs designated as orphan products by the Food and Drug Administration (FDA) treat patients with rare diseases but can also be approved to treat common diseases. Prozac (fluoxetine; Eli Lilly, Indianapolis, IN), for example, received an orphan designation from the FDA for the treatment of autism and body dysmorphic disorder in children and adolescents, but it is widely administered to treat depression. Access to orphan-designated products by vulnerable patient populations, whether for orphan indications or not, is facilitated by the federal 340B program that requires pharmaceutical manufacturers to provide these outpatient drugs at heavily discounted prices to hospitals and clinics that treat poor and underserved populations. As a result of a federal district court ruling in October 2015,^1^ however, pharmaceutical manufacturers are no longer required to discount orphan-designated products for certain eligible health care facilities. The decision may result in higher acquisition costs of orphan-designated products for these entities. This ruling may also embolden those seeking to challenge 340B Omnibus Guidelines, which were drafted in response to criticisms against 340B program inadequacies.^2^ It has been a revolving door of policy decisions made by the Department of Health and Human Services (HHS) requiring pharmaceutical manufacturers to discount drugs at certain 340B-eligible facilities and of pharmaceutical industry–initiated lawsuits that negate these HHS policy decisions. These implications call for further action from Congress to provide more clarity on the requirements for 340B program participation, which is central to whether 340B-eligible facilities will be able to purchase cancer drugs with orphan disease designations at discounted prices.

## Competing Considerations

Underlying the 340B ruling are two distinct policy considerations—encouraging pharmaceutical manufacturers to develop medications that treat rare disorders and providing discounted prescription drugs to entities that serve vulnerable populations.

The Orphan Drug Act, enacted in 1983, incentivizes the development of medications for rare diseases that affect fewer than 200,000 people in the United States or that affect more than 200,000 persons but without a reasonable expectation that the costs of development will be recovered through sale of the drug. Although the orphan designation is granted to medications that treat rare diseases, physicians frequently prescribe orphan-designated medications to treat common diseases. Since the law was passed in 1983, the proportion of new agents submitted to treat rare disorders and ultimately approved by the FDA has risen steadily.^3^ In calendar year 2015, the FDA approved 45 novel new drugs, and 47% (21 of 45) of those medications had at least one orphan disease indication.^4^ The expenditures for medications with an orphan indication (including both orphan-only and partial-orphan drugs) grew from $15 billion to $30 billion, representing 4.8% to 8.9% of total US drug expenditures from 2007 to 2013.^5^

Today, many orphan-designated medications are blockbusters because they face less competition through statutory protection and are often expensive biologic drugs with both orphan and nonorphan uses. Simply by receiving an orphan designation, manufacturers are granted longer market exclusivity for their drug. Seven of the top 10 best-selling drugs worldwide in 2015 had an FDA-approved orphan indication.^6^ Rituximab, now the twelfth all-time best-selling medication in the United States,^6^ for example, was first marketed for nonorphan conditions to treat lymphoma and rheumatoid arthritis.^7^ Studies were subsequently conducted in diseases with fewer individuals, and rituximab received an orphan indication to treat Wegener granulomatosis and microscopic polyangiitis, two rare disorders that cause vasculitis. This strategy of acquiring an orphan designation for drugs that also treat common diseases has significant potential for profit. The majority of drugs for indications with fewer than 10,000 patients in the United States are priced at or above $200,000 per year.^8^

Given the high pricing of drugs in America, Congress created the 340B program in 1992 to help uninsured, indigent patients gain better access to prescription medicines.^9^ To help achieve this goal, the program requires pharmaceutical manufacturers to provide front-end discounts, typically 30% to 50%, on outpatient prescription medicines to entities that serve high numbers of uninsured and indigent patients.^9^ The 340B program has stirred controversy, however, because it allows covered entities to purchase discounted drugs prescribed to all of their patients, including patients with Medicare or private insurance.^9^ The program also does not require covered entities to pass on cost savings to vulnerable patients, although some do.^9^ The Affordable Care Act of 2010 (ACA) intensified this controversy by expanding the types of covered entities to the following: children’s hospitals, free-standing cancer hospitals, critical access hospitals, rural referral centers, and sole community hospitals.^10^ However, to preserve pharmaceutical manufacturers’ incentive to develop medications treating rare disorders, all of these newly covered entities, except children’s hospitals, were excluded from discount pricing for orphan-designated products under the ACA (Orphan Exclusion).^10^

For years, concerns have been raised that the 340B program has moved beyond its original intent with greatly expanded hospital participation over time. The number of hospital organizations participating in 340B grew from 583 in 2005 to 1,365 in 2010, and to 2,170 as of January 2015.^2,11^ Although the growth has largely been in rural and cancer hospitals with ≤ 25 beds, the expanded list of covered entities under the ACA may have accentuated the controversy surrounding the 340B program and resulted in recent litigation limiting the scope of 340B related to the Orphan Exclusion.^1^

## Lawsuits and HHS Decisions

The 340B-covered entities and pharmaceutical manufacturers hold directly opposing views on the scope of the Orphan Exclusion—340B entities argue that the exclusion applies only when orphan-designated products are used for rare indications. Citing statutory text, pharmaceutical manufacturers counter that the exclusion applies even when orphan-designated products are used for nonorphan indications. The HHS took the position favored by 340B entities in its final rule issued in July 2013.

In September 2013, the Pharmaceutical Research and Manufacturers of America (PhRMA) brought suit against HHS challenging the validity of its final rule, claiming that HHS lacked legislative authority to issue the rule and that the rule contradicted underlying statutory language. In May 2014, the Court found in favor of PhRMA and stated that HHS had indeed exceeded the scope of its legislative authority in making the challenged rule.^12^ HHS responded by implementing its Orphan Exclusion policy as an interpretive (rather than a final) rule,^13^ effective July 2014. In October 2014, PhRMA again filed suit against HHS.^1^ PhRMA argued that the interpretative rule was actually a final rule and that the exclusion should be based on designation as an orphan product, regardless of whether it was used to treat rare disorders. On October 14, 2015, the District Court again sided with PhRMA.^1^

## What’s Next

The Court’s decision will affect the pricing of orphan-designated products for newly covered 340B entities that serve >10 million people nationwide.^9^ In particular, these hospitals can no longer purchase orphan-designated products at 340B discounted prices, even when these drugs are used for nonorphan indications. This ruling affects, for example, free-standing cancer clinics that routinely purchase expensive oncology biologics with orphan indications to the extent these biologics are used for nonorphan indications. Covered entities benefit from dispensing drugs covered under the 340B program because Medicare and private insurers pay for these drugs at a standard rate, even if hospitals are able to procure them at discounted prices.

Beyond its impact on the acquisition costs of orphan-designated products at newly covered entities, the Court’s decision may provide a roadmap for future challenges to HHS’s recently proposed 340B Omnibus Guidance,^14^ in which HHS aimed to reform many controversial aspects of the program. For example, the Omnibus Guidance clarifies that eligible patients include only those with an actual encounter with the covered entity, thereby limiting the use of the program. It also solves a potential problem of double discounts.^14^ Further, the Guidance includes new obligations for pharmaceutical manufacturers and better aligns the 340B program with the goal of serving vulnerable populations.^14^ Yet, because Congress did not provide HHS with rule-making authority with respect to 340B, the reasoning of the district court cases may allow pharmaceutical manufacturers to challenge the Omnibus Guidance, especially if it is construed as a final rule.

In conclusion, although it is possible to appeal the recent 340B ruling, the Court of Appeals is unlikely to overturn the ruling without action from Congress. Through unintended regulatory and statutory oversight, the recent court wins will allow manufacturers to increase providers’ acquisition costs of drugs ultimately used for large populations of patients. Manufacturers’ ability to charge undiscounted prices for nonorphan indications is contrary to the goals of the Orphan Drug Act to encourage drug development for rare diseases. High drug prices are a real concern—although pharmaceutical manufacturers have established over 200 different patient assistance programs^15^ that help provide financial assistance or free drugs to low-income individuals,^16-18^ these programs remain underutilized by targeted populations,^19,20^ primarily because of complex eligibility requirements and assistance guidelines, as well as time-consuming enrollment processes.^21^ In addition, pharmaceutical manufacturers may initiate further lawsuits challenging the Guidance as inconsistent with the 340B statute, jeopardizing reforms of the 340B program. These consequences result from both pharmaceutical manufacturers and 340B institutions exploiting regulatory gaps.

Therefore, Congress should consider amending the statute to clarify that the Orphan Exclusion is applicable only when these drugs are administered for rare indications. Congress should also provide HHS with additional rule-making authority to interpret the 340B statute and address other oversight issues that are the subject of continued debate between 340B covered entities and pharmaceutical manufacturers. Improved regulatory clarity coupled with greater government oversight^11^ of the 340B program will help advance the program’s goals and provide greater stability and rationality in the program’s future direction, given conflicting considerations among HHS, covered entities, and manufacturers.
